# Hydrogel mechanical properties in altered gravity

**DOI:** 10.1038/s41526-024-00388-2

**Published:** 2024-08-08

**Authors:** Vanja Mišković, Immacolata Greco, Christophe Minetti, Francesca Cialdai, Monica Monici, Arianna Gazzi, Jeremiah Marcellino, Yarjan Abdul Samad, Lucia Gemma Delogu, Andrea C. Ferrari, Carlo Saverio Iorio

**Affiliations:** 1 Centre for Research and Engineering in Space Technologies, École Polytechnique de Bruxelles, Université libre de Bruxelles, Brussels, Belgium; 2 ASAcampus Joint Laboratory, ASA Research Division, Department of Experimental and Clinical Biomedical Sciences « Mario Serio », University of Florence, Florence, Italy; 3 Department of Chemical and Pharmaceutical Sciences, University of Trieste, Trieste, Italy; 4 Department of Biomedical Sciences, University of Padua, Padua, Italy; 5 Cambridge Graphene Centre, University of Cambridge, Cambridge, CB3 0FA UK; 6 Department of Aerospace Engineering, Khalifa university of Science and Technology, Abu Dhabi, 127788 UAE; 7 Department of Biological Science, Khalifa university of Science and Technology, Abu Dhabi, UAE

**Keywords:** Biomedical materials, Biophysics

## Abstract

Exposure to altered gravity influences cellular behaviour in cell cultures. Hydrogels are amongst the most common materials used to produce tissue-engineering scaffolds, and their mechanical properties play a crucial role in cell-matrix interaction. However, little is known about the influence of altered gravity on hydrogel properties. Here we study the mechanical properties of Poly (ethylene glycol) diacrylate (PEGDA) and PEGDA incorporated with graphene oxide (GO) by performing tensile tests in micro and hypergravity during a Parabolic flight campaign, and by comparing them to the same tests performed in Earth gravity. We show that gravity levels do not result in a statistically significant difference in Young’s modulus.

## Introduction

Despite the emerging interest in the impacts of space travel on human health^1^, and therefore the influence of altered gravity on the biology of living systems^2^, its impact on tissue engineering is not well understood^3^.

Gravitational studies on cellular behaviour were carried out during space missions^4,5^, and in altered gravity achieved through the use of various on-ground facilities^6–11^. In vitro studies revealed alterations of fibroblasts^4,6^, keratinocytes^7^, endothelial^8,9,12^, mesenchymal^5^ and immune^10,11^ cells functionality. It has been observed alterations in transcription, translation, and organisation of cytoskeletal proteins, such as altered organisation of the microtubule network, and of the actin microfilaments, that result in morphological changes. Altered production of extracellular matrix proteins following microgravity conditions was also reported^4,8,13,14^. In both 2D and 3D cultured cells, their environment and choice of biomaterial are influencing cellular behaviour^15–18^. While most space-related studies focus on cellular changes in altered gravity^6–9^, its influence on biomaterial mechanics, diffusion and absorption properties is still unknown.

Hydrogels are a widely studied group of scaffolding materials in tissue engineering^19–23^ due to their hydrated polymer network, which allows them to absorb a large amount of fluids (orders of magnitude more than their own weight)^24^. In their swollen state, hydrogels provide cells with an environment that resembles that of the natural tissue^20,23^. The hydrogel mechanical properties and their role in the interaction with cells have been discussed in several reviews^20,25–27^ where the influence of stiffness/elasticity on cellular behaviour is highlighted. Therefore, possible changes in biomaterials induced by altered gravity should not be overlooked. The influence of gravity on gels is not well known, and existing studies mainly focus on gel synthesis in hyper and microgravity^28–31^.

We use Poly (ethylene glycol) diacrylate (PEGDA) and PEGDA-graphene oxide (PEGDA-GO) hydrogels. PEDGA is a synthetic material that has been tested in the tissue-engineering field mainly to enhance mechanical properties^32–34^ and increase the printability of bioinks^35^. PEGDA can be used to build three-dimensional scaffolds that give cells a place to grow, develop, and arrange themselves into tissues. These scaffolds can be created with appropriate mechanical and biochemical stimuli to direct cell behaviour and tissue development. PEGDA hydrogels are thus suitable for use in applications involving wound healing^36–39^ PEGDA macromers, owning to their acrylic groups located at both ends^34^, can be photopolymerized in the presence of photoinitiator^40^, which, when exposed to UV light, generates free radicals necessary to initiate polymerisation. Swelling and mechanical properties of PEGDA hydrogels can be modified and controlled by varying the total polymer ratio^41^, macromers’ molecular weight^41^ or concentration of photoinitiator^42^. However, PEGDA hydrogels alone do not provide an ideal environment for cells, mainly due to their lack of cell adhesion sites^34,43^. In previous studies, PEGDA networks have already been functionalized with cell adhesive moieties like arginine-glycine-aspartic acid (RGD)^44^ or crosslinked with extracellular matrix (ECM)-derived components^45–47^. These findings indicated that the addition of cell adhesive moieties promoted cell attachment and improved cell growth and differentiation^48^. In recent years, the application of biocompatible nanomaterials in tissue engineering, such as GO, has gained much attention in biomedical application^49^. Graphene oxide (GO) has attracted a lot of interest in the field of tissue engineering because of its special characteristics and potential uses. Because it is well tolerated by live cells and tissues, graphene oxide has demonstrated excellent biocompatibility. Although graphene oxide has a promising future in tissue engineering, issues still need to be resolved, such as long-term biocompatibility and potential toxicity at high concentrations^50^. Ref. ^43^ reported that incorporating PEGDA hydrogels with graphene oxide (GO) can enhance cell viability and survival.

Therefore, here we investigate the influence of altered gravity on PEGDA and PEGDA-graphene composites during the parabolic flight campaign. This allowed us to perform experiments in hypergravity (1.8–2 times higher than Earth’s gravity g_0_) and in microgravity (~10^−3^g_0_) on a platform more easily accessible than sounding rockets^51^, and International Space Station (ISS) flights^51^, while still allowing for relevant scientific outcomes^51^. We find that gravity has no effect on PEGDA and PEGDA-GO hydrogels’ mechanical properties. This will allow a better understanding of the origin of cellular changes happening in the space environment, by eliminating the possibility that some cellular changes are not caused by gravitational forces, but are the results of changes in hydrogels’ behaviour.

## Results and discussion

### Overview of the parabolic flight experiment

**Fig. 1:**
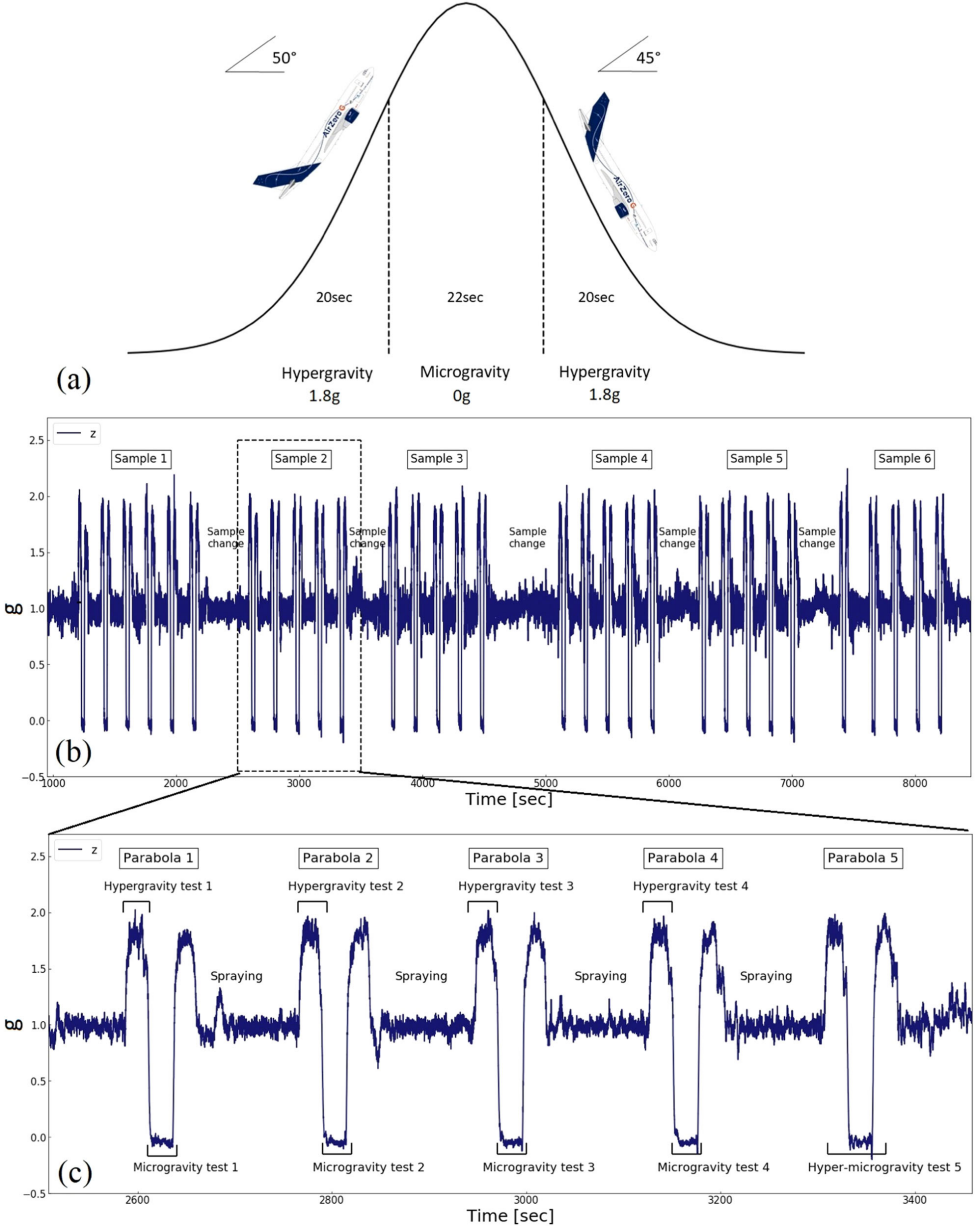
Parabolic flight manoeuvre. **a** Typical path of the aircraft during a single parabola. Adapted from ref. ^51^. **b** Parabolic flight z-axis acceleration data taken by the accelerometer inside the experimental test rig. **c** Timeline of the standard testing procedures registered during the flight.

The experiments were performed as a part of the 72nd European Space Agency (ESA) Parabolic flight campaign, conducted from Bordeaux-Mérignac Airport in France, on-board the Airbus A310 Zero Gravity (ZeroG) aircraft^51^. This follows a parabolic flight manoeuvre, Fig. 1 divided into 3 stages^51^: (1) pull-up; (2) parabolic trajectory, (3) pull-out. During pull-up, the plane is directed from a horizontal position upwards at an angle of 50**°**, and the gravity level (g) is 1.8–2.0g_0_^51^. This hypergravity phase lasts ~20s^51^. The aircraft follows a ballistic trajectory, and the weightless phase starts when the aircraft enters the parabolic trajectory, assuring a quasi-free fall state (~10^−3^g_0_^51^). This microgravity phase (µg) lasts ~22s^51^. The pull-out is the last stage of the manoeuvre^51^. The aircraft has a descending path at an angle of 45° (*g* ~ 1.8g_0_^51^). The duration and hypergravity conditions are symmetrical with respect to the pull-up phase.

Parabolas are repeated 31 times a day, resulting in ~11.5 mins of weightlessness. They are divided into 6 sets, each containing 5 parabolas, Fig. 1a. The testing protocol is designed to facilitate sample changes and assure that all safety procedures are followed. Two types of tests are done: (1) 1.8g_0_, µg_0_, and g_0_; (2) standard, as summarised in Table 1. The 1.8g_0_, µg_0_, g_0_ tests are done to compare the mechanical properties of hydrogels with 0%SR. These include one parabola for PEGDA and one for PEGDA-GO.

**Table 1:** Summary of all tests

Environment	Test type	Sample	Outcome
Parabolic flight test	1.8g_0_, µg_0_, and g_0_ test	PEGDA (dry) PEGDA-GO (dry)	Stress–strain curves for the same sample
	Standard test (Fig. 1b) without spray	PEGDA-GO (dry)	4 stress–strain curves in 1.8g_0_ and µg_0_. 1 stress–strain curve in 1.8g_0_ and µg_0_ that includes two gravity transitions.
	Standard tests with spray (Fig. 1b)	PEGDA and PEGDA-GO (dry and swollen)	4 stress–strain curves for dry PEGDA and 4 stress–strain curves for dry PEGDA-GO. 2 stress–strain curves for PEDA and 3 stress–strain curves for PEDA-GO with PBS in 1.8g_0_ and µg_0._ 2 stress–strain curves for PEGDA and 2 stress–strain curves for PEGDA-GO with PBS during 1.8g_0_ and µg_0_ that includes gravity transitions.
Ground test	Standard tests with spray	PEGDA and PEGDAGO (dry and swollen)	5 stress–strain curves for dry PEGDA and PEGDA with PBS in g_0_. 5 stress–strain curves for dry PEGDA-GO and PEGDA-GO with PBS in g_0_.

The standard test, described in Fig. 1b and Table 2 is done to study the influence of hyper and microgravity on hydrogels with different SR and to compare these with ground measurements. Within a set of 5 parabolas, 9 mechanical tensile tests are performed on the same hydrogel sample. In each of the first 4 parabolas, a tensile test is performed in both hypergravity and microgravity, with a testing speed of 2 mm/min and an increasing load up to 1.5 N. As soon as the maximum load is reached, the linear stages automatically return to the original position. During the 5th parabola, an experiment is performed for the entire duration of the manoeuvre, from hypergravity to microgravity, with the sample subject to an increasing load of up to 6.5 N. The test ends when the maximum load is reached or the sample ruptures. For each test, Young’s modulus (*E*) is calculated from the linear slope of the stress–strain curve.

**Table 2:** Summary of standard tests performed during one set of parabolas

Parabola	No. of tests/parabola	Procedure	Technical details
1 to 4	2 tests: 1 test in 1.8g_0_ 1 test in µg_0_	During each test, the sample is loaded up to max force. When this is reached, the sample is unloaded. The test starts and finishes in the same gravity.	Testing speed 2 mm/min. Force range: 0–1.5 N
5	1 test overall	The test starts at 1.8g_0_ and ends at µg_0_. The sample is loaded up to max force, and then unloaded. Curves include gravity transition.	Testing speed 2 mm/min Force range: 0–6.5 N

If not stated otherwise, tests start with 0%SR hydrogels and after every parabola 0.03 ml PBS is sprayed on it. This amount is chosen based on tests conducted on the ground, showing these hydrogels can absorb 0.03 ml/min PBS. The dimensions of the samples before spraying are measured using a mechanical caliper before placing the samples between the grips, while those of the wet samples are measured using the visualisation system, with software converting images’ pixels into mm.

## Methods

### Materials preparation

GO is prepared via a modified version of Hummer’s method^52^. A 9:1 ratio of concentrated H_2_SO_4_/H_3_PO_4_ (900:100) is used to disperse graphite flakes with the average lateral size 200–300 µm (Sigma-Aldrich) and dissolve KMnO_4_. The concentration of graphite is 1 wt% and that of KMnO_4_ 6 wt%. The suspension is heated to ~60 °C and stirred for >12 h. A diluted solution (~1000 ml) of H_2_O_2_ (0.25 wt%) in Deionized water (DIW) is then added. The suspension is cooled to room temperature (~21 °C) and then centrifuged at ~5000 rpm for 3 h. The remaining solid material is washed with DIW at least 10 times. Atomic force microscopy (Bruker Dimension Icon) shows a flake thickness ~1–2 nm, corresponding to ~2–3 GO layers^53^.

Hydrogels are produced with PEGDA (average molecular weight 700) and a photoinitiator, Irgacure 2959 (2-Hydroxy-4’-(2-hydroxyethoxy)-2-methylpropiophenone), both sourced from Sigma-Aldrich. Dulbecco’s Phosphate Buffered Saline (PBS) from Sigma-Aldrich is used for hydrogel swelling. PBS was chosen since it is amongst the most commonly chosen swelling media to model hydrogel behaviour in biological studies^54,55^.

Two sets of samples are prepared, Table 3. PEGDA is first dissolved in DIW at 40 w/v%. The photoinitiator is added to the PEGDA precursor solution at 1 w/v %. PEGDA-GO is then obtained by adding a GO water dispersion (2 wt%). All solutions are sonicated for 6 mins until they are homogeneous, then polymerised in a dog-bone shaped Teflon mould by UV irradiation for 9 mins using crosslinker AnalitikJena, an intensity 10 J/cm² at 365 nm. A time of 9 min was the minimum time in which PEGDAGO samples polymerised.

**Table 3:** Hydrogel samples

Sample	PEGDA (w/v %)	GO (w/v %)^a^	Photointiator (w/v %)	Time under UV (min)
PEGDA	40	0	1	9
PEGDA-GO	40	0.005	1	9

^a^With respect to PEGDA.

Once dried, the hydrogels are ~1.8 ± 0.025 mm thick, and 6.5 ± 0.049 mm wide. The swelling ratio is calculated as^56^:

1 $${SR}=\frac{{(w}_{s}-{w}_{d})}{{w}_{d}}\times 100 \%$$

where *w*_*d*_ is the dry hydrogel mass, and *w*_*s*_ is that after spraying PBS.

The stiffness *k* (N mm^−1^) and Young’s modulus *E* (MPa) are calculated as follows^57^:

2 $$\varepsilon =\frac{\triangle l}{{l}_{0}}=\frac{l-{l}_{0}}{{l}_{0}}$$

3 $$\sigma =\frac{F}{A}$$

4 $$k=\frac{F}{\triangle l}$$

5 $$E=\frac{\sigma }{\varepsilon }$$

Where *ε* (mm mm^−1^) is the strain, *l*_0_ (mm) and *l* (mm) are the initial and final length of the sample, *σ* (MPa) stress, *F* (N) is the measured force, and *A* sample’s cross-section (mm^2^).

### Hardware design

**Fig. 2:**
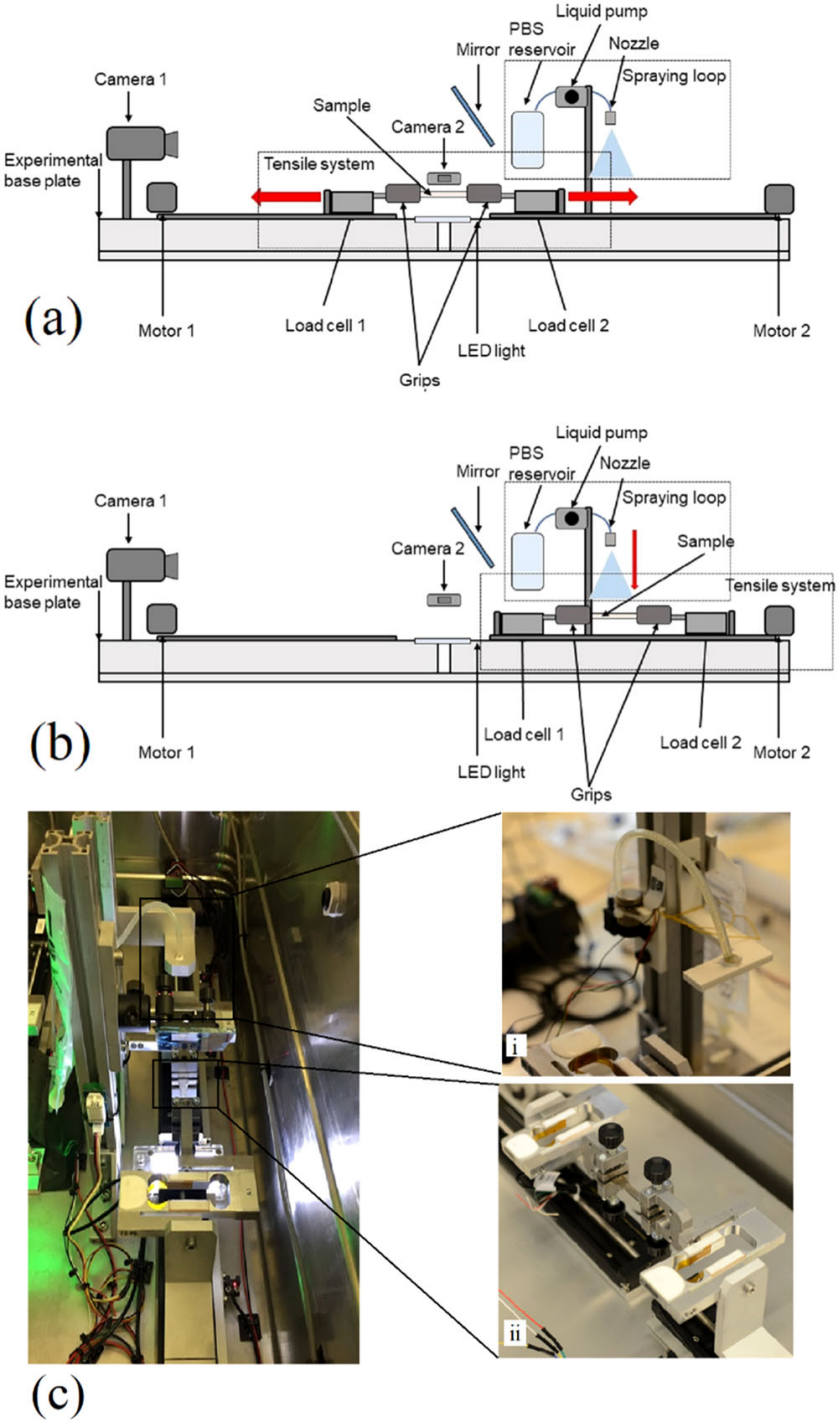
Schematic tensile system. **a** Sample in Position 1 for the tensile test, **b** sample in position 2 for spraying. **c** Experimental setup inside a protective box. **i**–**ii** detailed views of **i** spraying loop and **ii** tensile system.

The setup for hydrogel tensile tests is in Fig. 2. This comprises 3 main parts: (i) apparatus for generating tensile stress; (ii) spraying system to induce swelling; (iii) visualisation system.

The tensile apparatus has 2 linear translation stages with attached load cells, measuring the applied force. Samples are fixed between two grips so that the pulling force is applied from both sides. The spraying system consists of a PBS reservoir (plastic bag), a liquid diaphragm pump, commercial flexible silicone tubing with an external diameter of 3 mm and a 3 mm nozzle. Samples are sprayed with PBS to modify the hydrogel’s fluid content. The sample has two positions. Position 1 (Fig. 2a) for performing the tensile test and Position 2 for spraying (Fig. 2b). Spraying with PBS is introduced since it is proven that typical synthetic materials show a swelling-weakening behaviour, which always suffer from a sharp decline in mechanical strength after swelling because of the dilution of the network^55,58–61^. In this work, we tested hydrogels in different swelling conditions to investigate the influence of microgravity changes by increasing the swelling ratio of hydrogels. A visualisation system (digital cameras 1 and 2 and mirror) is used to observe the samples and measure the dimensions needed for stress and strain calculations (Eqs. 2–5). Digital camera 1, Fig. 2, is fixed on the same plate as the tensile system, in parallel with the sample. It is used to observe the test when the protective box is closed and measure the samples’ width after spraying. Camera 2 is fixed perpendicular to the sample, on the box wall, and used to measure the distance between the two grips, the sample length, and sample thickness.

### Discussion on hydrogels’ mechanical changes in microgravity

**Fig. 3:**
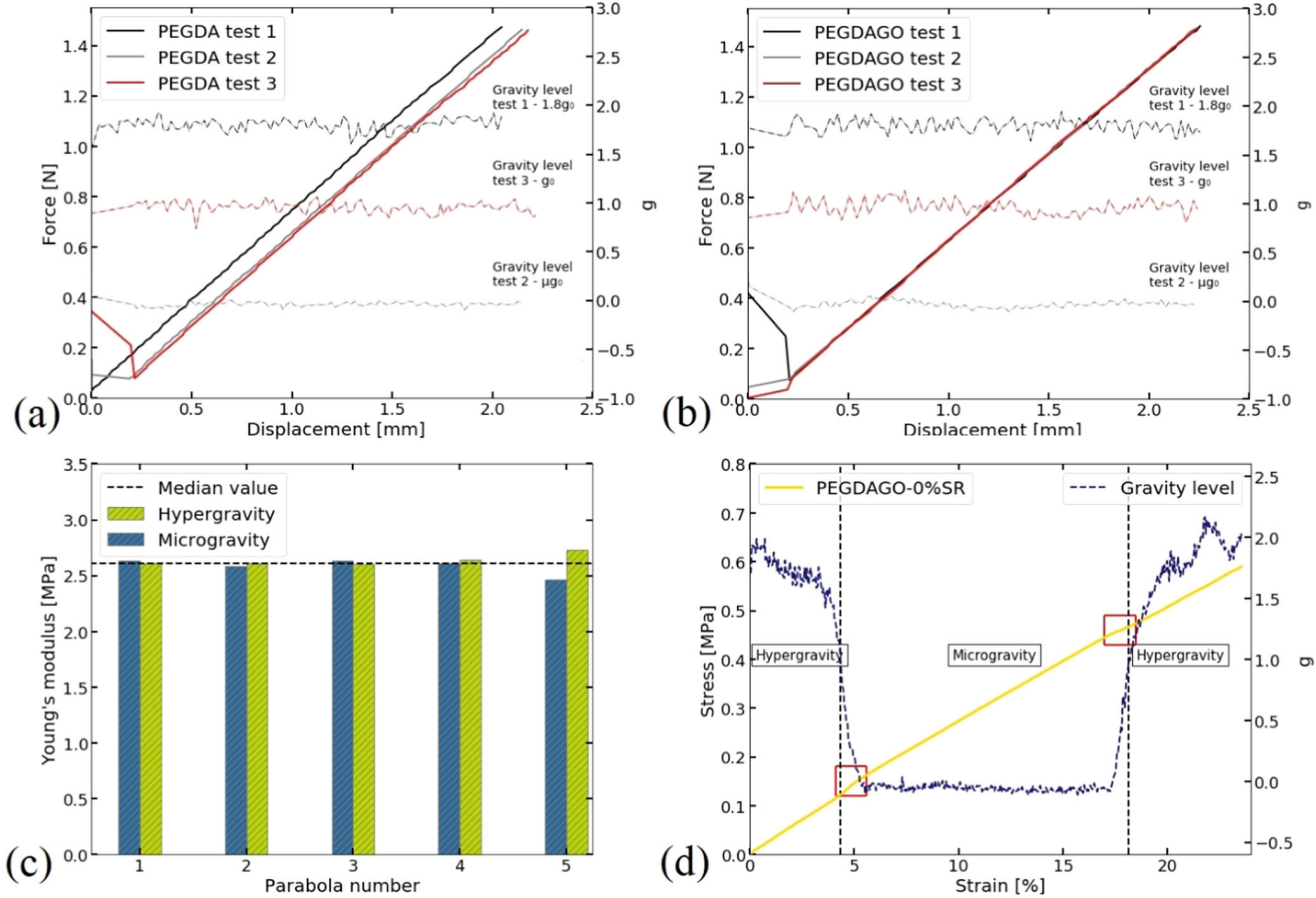
Force–displacement curves. **a** PEGDA curves; (**b**) PEGDA-GO in hyperg-, micro-, and Earth gravity curves; (**c**) *E* changes during 5 cycles in hyper- and microgravity; (**d)** Stress–strain curve in the 5th parabola, during hyper- and microgravity, including the transition between two gravities (red box). The test is stopped when a maximum force of ~6.5 N is reached.

To investigate the influence of gravity on the force–displacement, thus stress–strain, curve, a tensile test on the same sample is performed in hyper-, micro- and Earth gravity, for PEGDA (Fig. 3a) and PEGDA-GO (Fig. 3b) hydrogels in the elastic region, until a force of 1.5 N is reached. The statistical significance of differences between the samples is calculated using a two-tailed test (*t*-test) in SigmaPlot12.5 (Systat Software, San Jose, CA). A probability value (*p*), defined as a measure of the probability that an observed difference could have occurred just by random chance^40^, is used to compare samples from different groups. *p* < 0.05 is taken as significant since 0.05 is considered the standard threshold for statistical significance in biomedical studies^62^. Table 4 summarises all samples tested on the ground and on-board. Results in Table 5c are average values +− the standard error of the mean (SEM), defined as the standard deviation divided by the square root of the sample number^63^.

**Table 4:** Samples tested on the ground and during flight

	PEGDA			PEGDA-GO		
	Ground	Hypergravity	Microgravity	Ground	Hypergravity	Microgravity
0% SR	6	4	4	5	4	4
5% SR	6	2	2	5	3	3
8% SR	6	2	1	5	3	3
10% SR	6	2	2	5	3	3
13% SR	4	2	2	5	2	2

**Table 5:** Results of tensile test on-board

(a) Hydrogels 0%SR: comparison between 3 gravity levels						
Sample	*k* (N/mm)			*E* (MPa)		
	g_0_	µg_0_	1.8g_0_	g_0_	µg_0_	1.8g_0_
PEGDA	0.70	0.70	0.71	2.32	2.32	2.31
PEGDA-GO	0.69	0.69	0.68	2.62	2.63	2.60
**(b) PEGDA-GO 0% SR:** ***E*** **(MP) comparison between 1.8g**_**0**_ **µ g**_**0**_ **during five parabolas**						
**Gravity**	**Parabola 1**	**Parabola 2**	**Parabola 3**	**Parabola 4**	**Parabola 5**	
1.8g_0_	2.61	2.61	2.60	2.64	2.73	
µ g_0_	2.63	2.58	2.64	2.61	2.47	
**(c) Average** ***E*** **(MPa)** ± SEM: Comparison between tests ground and on-board						
**Sample**	**Gravity**	**0% SR**	**5% SR**	**8% SR**	**11% SR**	**13% SR**
PEGDA	g_0_	2.46 ± 0.10	2.26 ± 0.08	2.11 ± 0.11	2.07 ± 0.11	1.85 ± 0.09
	1.8g_0_	2.28 ± 0.22	2.07 ± 0.03	1.84 ± 0.01	1.71 ± 0.02	1.75 ± 0.07
	µg_0_	2.27 ± 0.22	2.07 ± 0.03	1.84 ± 0.00	1.74 ± 0.01	1.73 ± 0.02
PEGDAGO	g_0_	2.51 ± 0.04	2.16 ± 0.06	2.05 ± 0.05	1.98 ± 0.06	2.01 ± 0.08
	1.8g_0_	2.35 ± 0.09	1.87 ± 0.08	1.79 ± 0.15	1.73 ± 0.14	1.59 ± 0.29
	µg_0_	2.36 ± 0.09	1.90 ± 0.09	1.83 ± 0.15	1.75 ± 0.15	1.70 ± 0.16

The difference in the stiffness, when comparing tests in 1.8g_0_, µg_0_, and g_0_ is negligible, Table 5a. These results suggest that g does not influence mechanical properties in the elastic region when SR is 0%. Since the stiffness for the samples in the table Table 5a is nearly identical, we can conclude that the difference in Young Modulus is due to the dimensions of the different samples that are taken into account while calculating *E* (see Eqs. 2–5). In order to ensure that multiple tests performed on one sample are comparable, we dedicate one set of parabolas for the standard test on the PEGDA-GO with 0%SR. This includes exposing one PEGDA-GO to 4 loading cycles in the same condition, with a maximum force~1.5 N, in hyper and microgravity. The results are in Fig. 3c. The last parabola in a set is used to observe changes in the curve during the transition between different gravity levels and includes a tensile test that starts in hypergravity, with maximum force set to 6.5 N, Fig. 3d. The median *E* of all tests, Table 5b, combining hyper- and microgravity, is 2.61 MPa. A maximum offset of ~6% compared to the median is observed during the 5th parabola microgravity, with *E* ~ 2.47 MPa, presumably due to the fact that this test is performed until 6.5 N, including the transition between two gravities, Fig. 3c. Unlike the test on the 5th parabola, other *E* differences are negligible (<5%). In Fig. 3d, changes in the slope are marked with a red box. When transiting from hyper- to microgravity, *E* decreases from 2.73 to 2.47 MPa (~9.6%). The second transition from micro- to hypergravity results in another *E* decrease from 2.47 to 2.33 MPa (~5.6%). These variations in slope during the transition can be attributed to the abrupt changes in the acceleration and their influence on the load cells

**Fig. 4:**
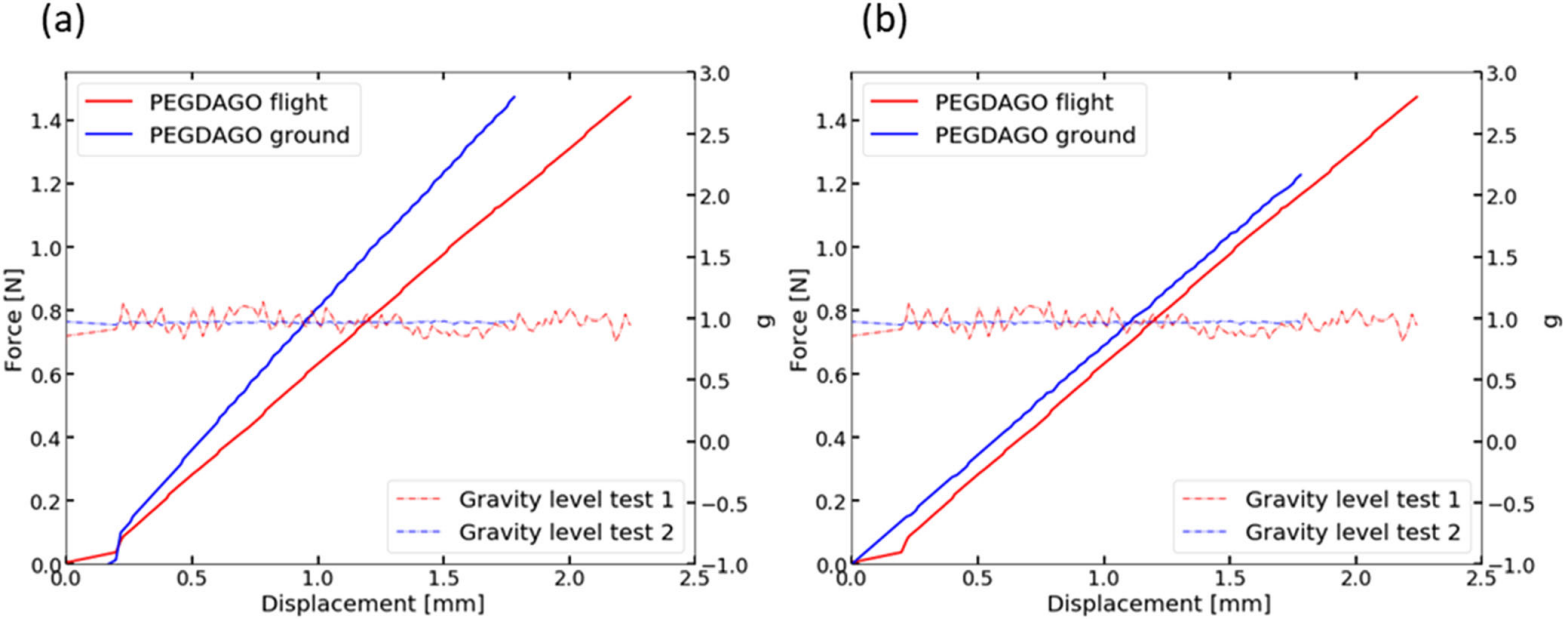
Force displacement curves. **a** Comparison between two hydrogels with 0%SR of the same size, tested on flight and on ground following the same procedure. **b** Same samples after the setup offset is calculated and applied to the ground data.

**Fig. 5:**
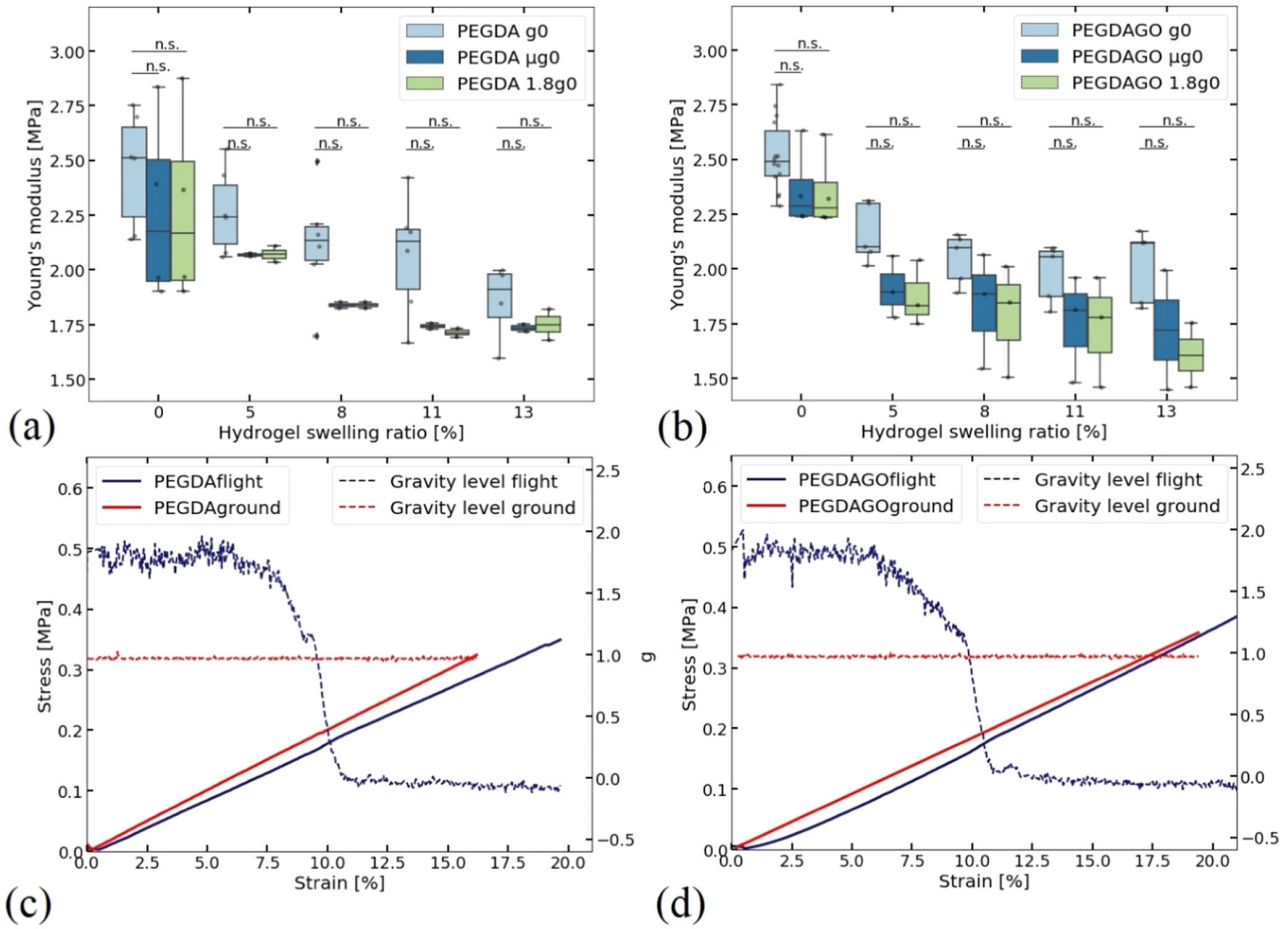
Mechanical properties of hydrogels under altered gravity conditions. *E modulus* (**a**) PEGDA and (**b**) PEGDA-GO hydrogels with different PBS content, comparing ground and flight data. The error bars indicate the minimum and maximum values. The line in the middle presents the median value. Outliners (anything higher or lower than 1.5 times the interquartile range) are presented with a diamond shape. *p < 0.05, n.s. = not significant (*p* > 0.05); Stress–strain curve of swollen (**c**) PEGDA and (**d**) PEGDAGO hydrogels. Comparison between ground and flight after the 4th spray. The test is stopped at a maximum force of ~6.5 N.

Raw data analysis for the tests conducted on the ground is in Fig. 4. The *E* comparison for all tests at Earth’s hyper and microgravity are in Fig. 5. Hyper and microgravity data come from identical samples, while tests on ground are performed on another batch. For both PEGDA and PEGDA-GO hydrogels, *E* decreases with increasing PBS content.

When comparing ground and flight tests for the same hydrogel type, the decreasing trend of *E* with PBS content increase is similar. Statistical analysis, see Methods, shows that there is no significant difference (*p* > 0.05) between ground, hyper and microgravity, except for PEGDA-GO after the first spray (*p* = 0.047). The fact that hydrogels with 0%SR have the same mechanical behaviour in the elastic region implies these differences are due to statistical error.

Further proof for this interpretation is obtained from a direct comparison between hydrogels with the same size, thus the same PBS uptake, after the 4th spray. Figure 5c, d compares ground and flight tests for PEGDA and PEGDA-GO after the 4th spray. For PEGDA, the difference between ground and microgravity is ~12.31% and ~8.83% for hypergravity. A different trend is observed for PEGDA-GO, with ~0.17% between ground and microgravity and~12.23% with hypergravity. We observe changes in slope during the gravity transition (Fig. 5a, b blue line), similar to those for the hydrogels with 0%SR. For PEGDA, Fig. 5c, the transition from hyper- to microgravity decreases *E* ~ 3.81%. For PEGDA-GO, Fig. 5d, transiting from hyper to microgravity, *E* increases ~13.7%.

Load cells are sensitive to abrupt gravity changes during tensile tests, including shifts between hyper and microgravity, and vice versa, Fig. 3b–d, as shown by changes in the slope of stress–strain curves between 3.8% and 13.7%. These changes do not have the same trend. Figure 3a, b show the lack of gravity effect on the hydrogels’ stiffness in the elastic region by comparing data from 3 different gravities. Considering our statistical analysis on tests done with hydrogels containing PBS (swelling ratio ~5, 8, 10, 13%), there is no significant change (*p* > 0.05) between tests in hyper, micro and Earth gravity, except for one point (out of 10 conditions). In all cases, *E* differences are due to the load cell response in gravity changes or small differences in material sizes and are not caused by changes in material behaviour. Further proof is obtained from Fig. 5c, d, where *E* differences for hydrogels with PBS, during the gravity transition are comparable to those for hydrogels with 0%SR.

The long-term stability of materials’ properties during microgravity is an aspect that should also be considered against the dynamical changes in the cellular arrangements and expressions that will also depend on the altered gravity conditions. Although at present it is not possible to draw conclusions, long-term experiments will allow for a better knowledge of this aspect and will be presented in further studies.

Finally, since experiments conducted during space missions are costly^64^, time-consuming^65^ and flight opportunities are scarce^64,65^, parabolic flights are useful for performing short experiments (the microgravity period of each parabola is ~22s^51^) in altered gravity in a cost-effective manner and provide an excellent opportunity to test instrumentation and phenomena prior to space flights^51^.

We found no correlation between gravity level and hydrogels’ mechanical properties in the elastic region. This conclusion is well-founded for hydrogels with 0%SR, and we believe it can be transferred for hydrogels swollen in PBS (swelling ratio ~5, 8, 10, 13%). Our experimental setup can be used to develop a database of mechanical properties in altered gravity and adapted for biological experiments where cells are seeded on/inside the hydrogels.

### Reporting summary

Further information on research design is available in the Nature Research Reporting Summary linked to this article.

### Supplementary information

Reporting Summary

## Data Availability

The authors confirm that the data supporting the findings of this study are available within the article and its supplementary materials. Raw data generated during this study are available from the corresponding author CSI on request.

## Code availability

For data collection, we used a custom code developed using C++. Accession codes will be available before publication. Data was visualised using Matplotlib and Seaborn libraries in Python 3.6.
