# Author Correction: Hydrogel mechanical properties in altered gravity

**DOI:** 10.1038/s41526-024-00426-z

**Published:** 2024-08-21

**Authors:** Vanja Mišković, Immacolata Greco, Christophe Minetti, Francesca Cialdai, Monica Monici, Arianna Gazzi, Jeremiah Marcellino, Yarjan Abdul Samad, Lucia Gemma Delogu, Andrea C. Ferrari, Carlo Saverio Iorio

**Affiliations:** 1 Centre for Research and Engineering in Space Technologies, École Polytechnique de Bruxelles, Université libre de Bruxelles, Brussels, Belgium; 2 ASAcampus Joint Laboratory, ASA Research Division, Department of Experimental and Clinical Biomedical Sciences « Mario Serio », University of Florence, Florence, Italy; 3 Department of Chemical and Pharmaceutical Sciences, University of Trieste, Trieste, Italy; 4 Department of Biomedical Sciences, University of Padua, Padua, Italy; 5 Cambridge Graphene Centre, University of Cambridge, Cambridge, CB3 0FA UK; 6 Department of Aerospace Engineering, Khalifa university of Science and Technology, Abu Dhabi, 127788 UAE; 7 Department of Biological Science, Khalifa university of Science and Technology, Abu Dhabi, UAE

**Keywords:** Biomedical materials, Biophysics

Correction to: *npj Microgravity* 10.1038/s41526-024-00388-2, published online 08 August 2024

In this article the affiliation, “Department of Aerospace Engineering, Khalifa University of Science and Technology, Abu Dhabi 127788, UAE” for the author “Yarjan Abdul Samad” was inadvertently omitted. The original article has been corrected.

